# Inoculation Effect of Methanotrophs on Rhizoremediation Performance and Methane Emission in Diesel-Contaminated Soil

**DOI:** 10.4014/jmb.2301.01007

**Published:** 2023-04-14

**Authors:** Ji Ho Lee, Hyoju Yang, Kyung-Suk Cho

**Affiliations:** Department of Environmental Science and Engineering, Ewha Womans University, Seoul 03760, Republic of Korea

**Keywords:** Methane, methanotroph, *Methylocystis*, *Methyloversatilis*, diesel-contaminated soil, rhizoremediation

## Abstract

During the rhizoremediation of diesel-contaminated soil, methane (CH_4_), a representative greenhouse gas, is emitted as a result of anaerobic metabolism of diesel. The application of methantrophs is one of solutions for the mitigation CH_4_ emissions during the rhizoremediation of diesel-contaminated soil. In this study, CH_4_-oxidizing rhizobacteria, *Methylocystis* sp. JHTF4 and *Methyloversatilis* sp. JHM8, were isolated from rhizosphere soils of tall fescue and maize, respectively. The maximum CH_4_ oxidation rates for the strains JHTF4 and JHM8 were 65.8 and 33.8 mmol·g-DCW^-1^·h^-1^, respectively. The isolates JHTF4 and JHM8 couldn't degrade diesel. The inoculation of the isolate JHTF4 or JHM8 significantly enhanced diesel removal during rhizoremediation of diesel-contaminated soil planted with maize for 63 days. Diesel removal in the tall fescue-planting soil was enhanced by inoculating the isolates until 50 days, while there was no significant difference in removal efficiency regardless of inoculation at day 63. In both the maize and tall fescue planting soils, the CH_4_ oxidation potentials of the inoculated soils were significantly higher than the potentials of the non-inoculated soils. In addition, the gene copy numbers of *pmoA*, responsible for CH_4_ oxidation, in the inoculated soils were significantly higher than those in the non-inoculated soils. The gene copy numbers ratio of *pmoA* to 16S rDNA (the ratio of methanotrophs to total bacteria) in soil increased during rhizoremediation. These results indicate that the inoculation of *Methylocystis* sp. JHTF4 and *Methyloversatilis* sp. JHM8, is a promising strategy to minimize CH_4_ emissions during the rhizoremediation of diesel-contaminated soil using maize or tall fescue.

## Introduction

Rhizoremediation is a promising technology that uses plants and rhizobacteria to remediate contaminated soil with total petroleum hydrocarbons (TPHs) and heavy metals [1, 2]. TPHs containing various hydrocarbon compounds remain in soil for a long time and are harmful to soil organisms and contaminate surface water or groundwater [3, 4]. To efficiently remediate soil contaminated with TPHs, rhizoremediation technologies using tall fescue and maize have been proposed [5-8]. TPHs can ultimately be mineralized into carbon dioxide and water by rhizobacteria in rhizosphere of plants [9]. However, metabolic intermediates may accumulate in soil depending on environmental conditions during the remediation of TPHs-contaminated soil [10]. In particular, methane (CH_4_) is emitted by anaerobic metabolism of TPHs when there is insufficient oxygen in soil [7, 11]. The global warming potential of CH_4_ is 28 times higher than that of carbon dioxide [12]. According to the previous research, climate change impacts such as hot extreme weather and flood have occurred more frequently than previous, and the researchers confidently suggested that human activity is responsible for these changes [13]. Therefore, efforts to mitigation CH_4_ emissions are required to respond to climate change.

Until now, most of research on rhizoremediation of TPHs-contaminated soil have focused on TPHs removal efficiency. However, in order to respond to climate change, technology development is required to remove TPHs while minimizing CH_4_ emissions. To minimize CH_4_ emissions during the rhizoremediation of TPHs-contaminated soil, the utilization of CH_4_-oxidizing bacteria is one of useful strategies [6, 7, 14]. Studies have been conducted to reduce CH_4_ emissions in landfills, agricultural land and hydropower reservoir using CH_4_-oxidizing consortia [15-19], but there are few cases of using CH_4_-oxidizing bacteria to reduce CH_4_ emissions in the process of remediation of TPHs-contaminated soil [20]. CH_4_-oxidizing bacteria that can be used to reduce CH_4_ emission in the TPHs-contaminated soil remediation should have CH_4_-oxidizing activity in the soil system, and should not inhibit the activity of bacteria responsible for TPHs degradation. Therefore, if methane-oxidizing pure bacteria that do not inhibit the activity of TPHs-degrading microorganisms are applied, methane emissions can be mitigated without adversely affecting the remediation efficiency of TPHs-contaminated soil.

In this study, CH_4_-oxidizing rhizobacteria were isolated from the rhizospheric soil of tall fescue and maize in TPHs-contaminated soils. The inoculation effect of the isolated bacteria on TPHs removal and soil CH_4_ oxidation potential was evaluated during the rhizoremediation of TPHs-contaminated soil. In addition, the behavior of the inoculated bacteria in the soil was monitored using a quantitative polymerase chain reaction (qPCR) method of the particulate CH_4_ monooxygenase gene (*pmoA*) [7, 20].

## Materials and Methods

### Enrichment Culture and Isolation of CH_4_-Oxidizing Rhizobacteria

Each soil sample (60 g) obtained from the rhizosphere of tall fescue (*Festuca arundinacea*) and maize (*Zea mays*) was suspended in 100 ml of a nitrate mineral salt (NMS) medium in 1.2-L serum bottle. The NMS medium contained; MgSO_4_·7H_2_O, 1 g; CaCl_2_, 0.2 g; KNO_3_ 1 g; KH_2_PO_4_, 0.272 g; Na_2_HPO_4_·12H_2_O, 0.717 g; Trace element solution 0.25 ml; 3,000 μM Cu^2+^ solution 10 ml in 1 L of distilled water. Trace element solution contained; FeSO_4_·7H_2_O, 200 mg; ZnSO_4_·7H_2_O, 10 mg; MnCl_2_·4H_2_O 3 mg; H_3_BO_3_, 30 mg; CoCl_2_·6H_2_O, 20 mg; CaCl_2_·2H_2_O, 1mg; NiCl_2_·6H_2_O, 2 mg; Na_2_MoO_4_·2H_2_O, 3 mg in 1 L of distilled water. Trace element solution was filtered by 0.45 μm filter before use. The NMS medium and 3,000 μM Cu^2+^ solution was autoclaved before use.

Each soil suspension was added to a 1.2-L serum bottle and then sealed with a butyl rubber stopper after purging with N_2_ gas to maintain anaerobic conditions. CH_4_ gas (99.99 %, Dong-A Specialty Gases Co., Republic of Korea) was injected into the bottle via a syringe to adjust the final headspace concentration to 5 % (v/v). Each serum bottle was incubated at 30°C under agitation at 150 rpm. The CH_4_ concentration was monitored periodically using a gas chromatograph (Agilent 7890A, USA) equipped with a flame ionization detector and a capillary column (30 m × 0.320 mm × 0.25 μm, HP5, Agilent, USA). N_2_ gas was used for carrier gas, and the temperature of the oven, inlet, and detector was 100, 210, and 300°C, respectively. The split ratio was 50:1, and the column flow rate was 75 ml·min^-1^.

When the CH_4_ concentration in the headspace of the bottle decreased below 50 ppm, additional CH_4_ gas was injected into the bottle to a headspace concentration of 5% (v/v). If CH_4_ oxidation was not observed for more than 2 days due to lack of oxygen, nitrogen and phosphorus during the enrichment culture, each serum bottle was opened in the clean bench and aerated for two hours. In order to resupply nitrogen and phosphorus, l ml of nitric acid concentrate solution (KNO_3_, 2 g·l^-1^) and 1 ml of phosphoric acid concentrate solution (KH_2_PO_4_, 0.52 g·l^-1^, Na_2_HPO_4_·12H_2_O, 1.65 g·l^-1^) was injected. After then, each serum bottle was sealed with a butyl rubber cap, and CH_4_ was injected by the same method. After incubating for 35 days, each 60 ml of the 1^st^ enriched culture was transferred to a new bottle and added with 60 ml of fresh NMS medium, and then each bottle was incubated for 12 days in the same manner as above. Each 70 ml of the 2^nd^ enriched culture was transferred into 70 ml of fresh NMS medium in a new bottle, and then incubated for 20 days in the same manner as above.

Each 3^rd^ enriched culture from the rhizosphere soils of tall fescue or maize (tall fescue or maize consortia) was serially diluted with the NMS medium and spread on the NMS-agar plates where 15 g·l^-1^ of agar is added in the NWS medium. The plates were incubated in an aerobic jar containing 5% (v/v) CH_4_, at 30°C for 14 days. Eight colonies on each NMS-agar plate were chosen, with each colony inoculated into 20 ml of NMS medium, with 5%(v/v) CH_4_, in a 120-ml serum bottle. Each strain exhibiting superior CH_4_-oxidizing activity from the tall fescue and maize consortia was selected and designated JHTF4 and JHM8, respectively.

### Identification of the Isolates

To identify the strain JHTF4 and JHM8, its colony on the NMS agar plate was picked and added into 100 μl of distilled water using a sterile loop. After then, the cell suspension was heated at 95°C and centrifuged to obtain its DNA. After PCR using the DNA template and 340F and 805R primer targeting 16S rRNA [21, 22], the sequences of the PCR products were analyzed by Macrogen (Republic of Korea). The resulting sequences were analyzed using the Basic Local Alignment Search Tool (BLAST) developed by the National Center for Biotechnology Information (NCBI). The sequences of the strain JHTF4 and JHM8 were deposited into the NCBI GenBank database under accession number MZ045833 and ON573373, respectively. A phylogenetic tree was constructed with the 16S rRNA sequences of the strain MZ045833 and ON573373, respectively. The strain JHTF4 and JHM8 was known as Methylocyctis sp. and *Methyloversatilis* sp. using the MEGA software (version 11, www.megasoftware.net) and the neighbor-joining algorithm.

To confirm whether each isolate was a pure culture, the following tests were conducted. Each culture broth cultivated in the NMS-CH_4_ medium, where CH_4_ was supplied as a sole carbon and energy source, was spread on an LB-agar plate to confirm whether heterotrophic bacteria were contaminated. In addition, after diluting the culture broth with sterile water, the diluted solution was spread on the NMS-agar plate, the plate was incubated in an aerobic jar containing 5% (v/v) CH_4_, at 30°C for 14 days. Ten colonies grown on the NMS-agar plate were randomly selected, and their 16S rRNA sequences were analyzed to determine whether they were identical to the nucleotide sequences of each isolate.

### Characterization of CH_4_ Oxidization by the Isolates

Each isolate was pre-grown in a 1.2-L serum bottle, containing 300 ml of NMS medium and 5% (v/v) CH_4_ at 30°C for 10 days. Each 20 ml of the pre-cultured broth (OD_600_ = 1.5) was transferred into a 600-ml serum bottle. CH_4_ gas was injected to be final concentration of 1, 5, 10, 15, or 20% (v/v) in the headspace of each bottle. Using Henry’s constant (20°C, 1 atm) for CH_4_, the total CH_4_ amount in each bottle is corresponded to 250, 1248, 2496, 3743, or 4991 mmole/bottle, respectively [23]. Using Henry’s constant for CH_4_, each CH_4_ concentration in the liquid is calculated as 14, 69, 139, 208, and 277 μM, respectively [23]. The rates of CH_4_ oxidation were calculated from the slopes of plots of CH_4_ concentration versus time. The maximum CH_4_ oxidation rate (*V*_max_) and saturation constant (*K*_m_) were determined using a Lineweaver-Burk plot [24]. Cell concentrations were determined using the relationship between the optical density measured at 600 nm and dry cell weight (DCW). The optical density was measured at 600 nm using a Libra S22 spectrophotometer (Biochrom, UK). Each experiment was performed in triplicate.

### Effect of Root Exudate on CH_4_ Oxidization by the Isolates

The effect of root exudate on CH_4_ oxidation rates by the strains JHTF4 and JHM8 was evaluated as follows. The root exudate of tall fescue and maize was prepared in a similar manner to that described in a previous paper [25]. Two concentrations of root exudate were prepared for each plant: The total organic carbon (TOC) concentrations of the maize root exudate were 126 and 252 mg·l^-1^, and the TOC concentrations of the tall fescue root exudate were 43, 85, and 213 mg·l^-1^. The chemical compounds in the NMS medium were also added to each root exudate at the same concentrations as in the NMS medium to produce the root exudate medium. Eight mL of the strain JHTF4 or strain JHM8 pre-culture broth was added to a 600-ml serum bottle containing 12 ml of the root exudate medium. For the control, the pre-culture broth was added into the NMS medium. CH_4_ gas was injected to be final concentration of 5% (v/v) in the headspace of each bottle. All experiments were performed at 30°C with 150 rpm in triplicate. The CH_4_ oxidation rate for each experimental condition was evaluated in a same manner as described above.

### Preparation of Pot Experiments

Rhizoremediation of diesel-contaminated soil was conducted on the rooftop of the New Engineering Building at Ewha Womans University, Seoul, Republic of Korea (37°57’ N, 126°95’ E). Soil, collected from the rooftop garden, was sieved with a 2-mm sieve to remove weeds and stones. The soil texture was loamy sand. After the soil was artificially contaminated with diesel at initial concentrations of 10,000 mg-diesel·kg-soil^-1^, the contaminated soil was then placed for 1 week. The soil was mixed manually once a day for 1 week. Compost (Seokgang Green Fertilizer Inc., Republic of Korea) was added to the contaminated soil to be final concentration of 5 % (w/w) to provide N and P [20].

The strains JHTF4 and JHM8 were cultured in the NMS medium supplemented with 5 % CH_4_, and each 20 ml of the culture broth (OD_600_ = 1.5) was added into 2 kg of the diesel-contaminated soil sample. A drainer and coarse sand were first placed on the bottom of each pot (diameter of 180 mm; height of 150 mm), and each inoculated soil with the strain JHTF4 or JHM8 was then added to each pot. As control, 2 kg of the contaminated soil without inoculation was added to a pot. Ten tall fescue or five maize seedlings were planted in each pot. The pot experiment was conducted in triplicate. The tall fescue seedlings were cultivated from seed for 45 days in a garden on the rooftop of the New Engineering Building at Ewha Womans University. Maize seedlings were purchased (Mojong 114, Republic of Korea), and cultivated for 45 days in the garden. The pot experiment was conducted on the rooftop of the New Engineering Building, Ewha Womans University for 63 days (May 24 to July 26, 2021). The pots were watered periodically to keep the plant from wilting during experiment.

### Inoculation Effect of the Isolates JHTF4 and JHM8 on Diesel Removal and Soil CH_4_-Oxidation Potential

To evaluate the inoculation effect of the isolates JHTF4 and JHM8 on diesel removal and soil CH_4_-oxidation potential during the rhizoremediation of diesel-contaminated soil, soil samples were taken randomly from each pot on days 0, 35, and 63. To analyze the residual diesel concentrations, the collected soil was freeze-dried, and 3 g of each sample was consequently added to individual test tubes, after which 10 ml of hexane-acetone (1:1, v/v) solution was added as the solvent for extraction. The diesel concentration was measured using the same method and gas chromatograph (GC 6980N system, Agilent Technologies, USA) as described in a previous study [20].

To measure soil CH_4_ oxidation potential, after air-drying the collected soils at room temperature, 2 g of each soil sample was added to a 600-ml serum bottle containing 12 ml of the NMS medium. The CH_4_ oxidation activity was evaluated in the same manner as described in Section 2.1.

To monitor the *pmoA* gene abundance in the diesel contaminated soil, qPCR was performed. The 16S rRNA gene was quantitatively evaluated for total bacteria abundances using 340F and 805R primer sets [20, 22]. The *pmoA* gene abundance was measured using A189f/mb661r [20, 26]. The ratio of *pmoA* gene to 16S rRNA gene was calculated to evaluate the relative abundance of CH_4_-oxidizing bacteria to total bacteria.

### Statistical Analysis

Microsoft Excel 2013 (Microsoft Co., USA) was employed to conduct *t*-tests and multiple comparisons with a *p*-value of 0.05 used to indicate a significant difference.

## Results and Discussion

### Identification and CH_4_ Oxidation Activity of the Isolates JHTF4 and JHM8

**Fig. 1:**
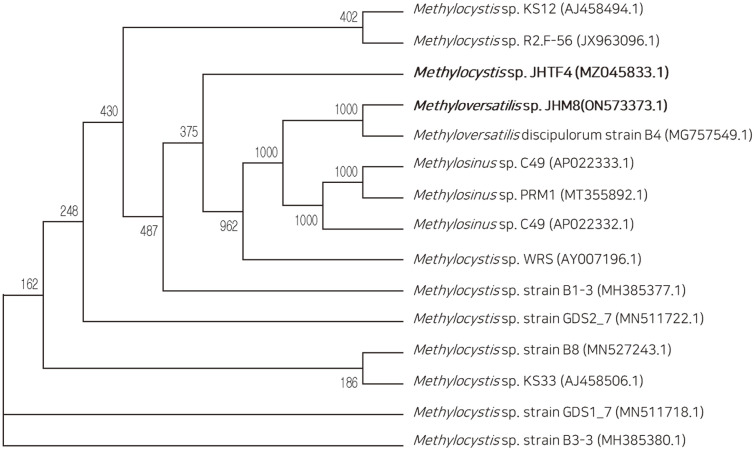
Phylogenic tree of the strains JHTF4 and JHM8 isolated from rhizosphere soils of tall fescue and maize, respectively. Boostrap values (percentages of 1,000 replications) are shown at the branch points. The scale bar represents 0.1 sub-stitutions per site. The 16S rRNA gene sequence of methantotrophs similar to the isolates were used as out-group.

The isolates JHTF4 and JHM8 from the rhizosphere soil samples planted with tall fescue and maize consortia were identified as *Methylocystis* sp. and *Methyloversatilis* sp., respectively (Fig. 1). Type I and type II methanotrophs have been differentiated by physiological characteristics [27]. *Methylocystis* spp., type II methanotrophs, assimilate formaldehyde by relatively less-productive pathway serine pathway [27]. *Methylocystis* spp. had been isolated form landfill cover soil, wetland, paddy soil, river, lake and so on [23, 28-31]. *Methyloversatilis* spp., type I methnotrphs, use the ribulose monophosphate pathway for formaldehyde assimilation [27]. *Methyloversatilis* spp. had been isolated from lake sediment, biofilm on steel-bentonite interphase, and so on [32, 33].

**Fig. 2:**
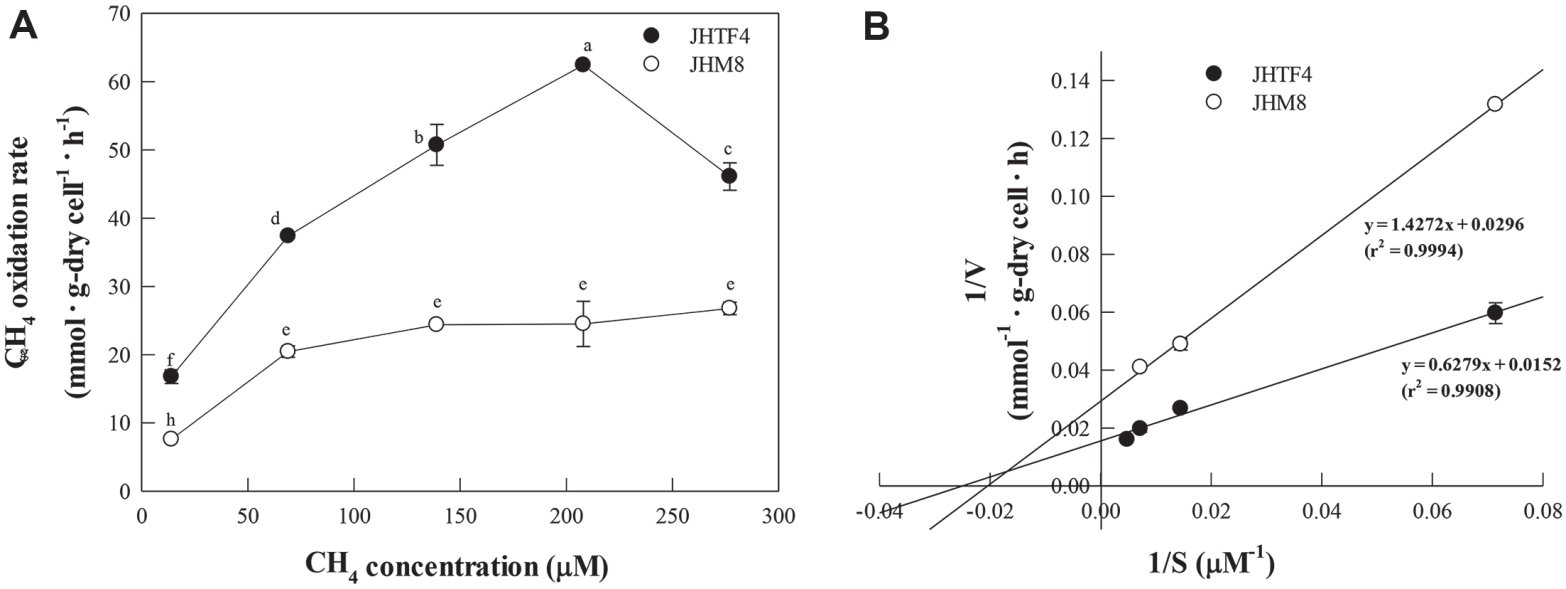
CH_4_ oxidation rates of the isolates JHTF4 and JHM8. (A) CH_4_ oxidation rates at different CH_4_ concentrations (B) Lineweaver-Burk plot of calculating the CH_4_ oxidation rates (*V*_max_) and saturation constant (*K*_m_).

Fig. 2 shows the specific CH_4_ oxidation rates of *Methylocystis* sp. JHTF4 and *Methyloversatilis* sp. JHM8 under different CH_4_ concentration (10,000-200,000 ppm). Both strains completely oxidized CH_4_ without a lag phase under the CH_4_ concentration of 50,000 ppm or less. They also completely oxidized 100,000 ppm CH_4_ after short lag period of 4-8 h (data not shown). In the high concentration CH_4_ condition of 150,000 ppm or more, CH_4_ was oxidized after a lag phase of 8-12 h, and 60-90% of initial CH_4_ was oxidized for 57 h. The specific CH_4_ oxidation rates of strain JHTF4 were increased with increasing initial CH_4_ concentration until 150,000 ppm CH_4_ (208 μM in liquid phase), but the rate decreased at 277 μM CH_4_ in the liquid (Fig. 2A). However, the specific CH_4_ oxidation rates of strain JHM8 were increased until 50,000 ppm CH_4_, and the rates were similar in the conditions from 50,000 to 200,000 ppm CH_4_ (Fig. 2A). At all CH_4_ concentrations, the CH_4_ oxidation rates of strain JHTF4 were superior to those of strain JHM8. Based on the Lineweaver-Burk plot (Fig. 2B), maximum CH_4_ oxidation rate (*V*_max_) and saturation constant (*K*_m_) for strain JHTF4 were calculated as 65.8 mmol·g-DCW^-1^·h^-1^ and 41 μM, respectively. For strain JHM8, *V*_max_ and *K*_m_ were 33.8 mmol· g-DCW^-1^·h^-1^ and 48 μM, respectively. *Methylocystis* sp. M6 had isolated from a landfill cover soil, and its *V*_max_ and *K*_m_ were reported as 4.93 mmol·g-DCW^-1^·h^-1^ and 23 μM, respectively [23]. The CH_4_ oxidation rates of strain JHTF4 and strain JHM8 were 13 and 7 times that of strain M6, respectively.

### Effect of the Root Exudate on CH_4_ Oxidation Activity

Rhizoremediation is a plant-assisted bioremediation using rhizobacteria, and has been applied in various ways to remediate soil or water contamination [34]. Rhizobacteria are influenced not only by physicochemical factors such as water content, pH, temperature, oxygen concentration, and nutrients, but also by root exudates secreted from plants [35]. Root exudates contain various organics and inorganics, and can act as a carbon source or growth factor for rhizosphere bacteria [36-38]. Therefore, root exudates can affect rhizosphere bacterial metabolism and alter the biogeochemical cycling of carbon and nitrogen in soil ecosystems [39]. Maize and tall fescue are often used in rhizoremediation processes, and they grow well in harsh or various environments [6, 7, 20, 40, 41].

**Fig. 3:**
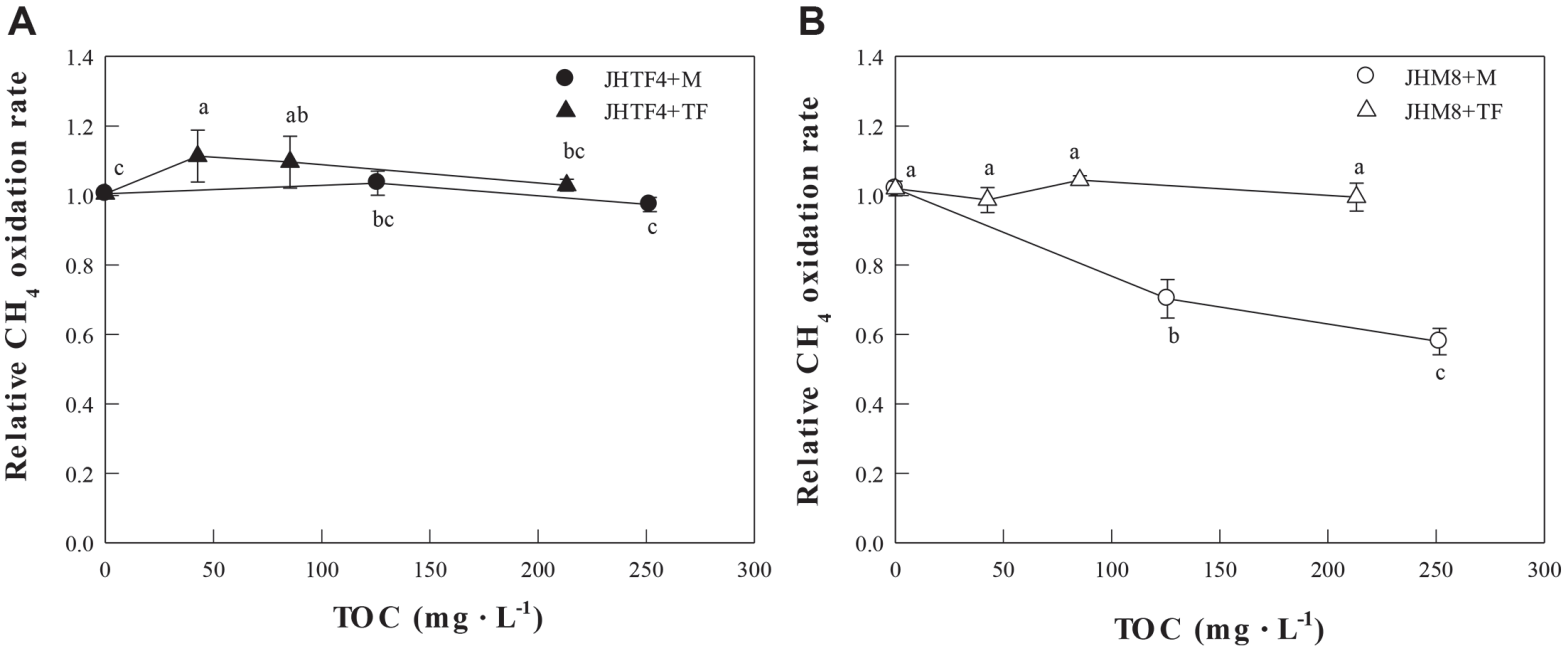
Effect of root exudate on CH_4_ oxidation activity of the isolates JHTF4 (A) and JHM8 (B). M, Maize root exudate; TF, Tall fescue root exudate.

Fig. 3 shows the addition effects of the root exudates of maize and tall fescue on the CH_4_ oxidation rates of strains JHTF4 and JHM8. Although the CH_4_ oxidation rate of strain JHTF4 slightly increased by the addition of root exudates below 100-150 mg-TOC·L^-1^, the CH_4_ oxidation activity of strain JHTF4 was hardly affected by the addition of root exudates (Fig. 3A). The CH_4_ oxidation rate of strain JHM8 was not affected by adding the root exudate of tall fescue, but it decreased with increasing the root exudate concentrations of maize (Fig. 3B). The addition effect of maize and tall fescue root exudates on the CH_4_ oxidation rate of the CH_4_-oxidizing consortium was evaluated [25]. As a result, the CH_4_ oxidation rate was slightly decreased by the exudate addition. It was considered that the activity of methanotrophs, which use C1 compound such and CH_4_ and methanol as a substrate, was inhibited by the carbon source contained in the root exudates [25]. In this study, the CH_4_ oxidation ability of the strain JHTF4 isolated from tall fescue rhizosphere was not inhibited by addition of maize root exudate as well as tall fescue. However, the CH_4_ oxidation ability of the strain JHM8 isolated from maize rhizosphere was not affected by tall fescue root exudate, but it was inhibited by maize root exudate. In order to interpret these findings in detail, further study on qualitative and quantitative analysis of the components of maize and tall fescue exudates is needed.

### Inoculation Effect of the Isolates JHTF4 and JHM8 on Diesel Degradation during Rhizoremediaiton

**Fig. 4:**
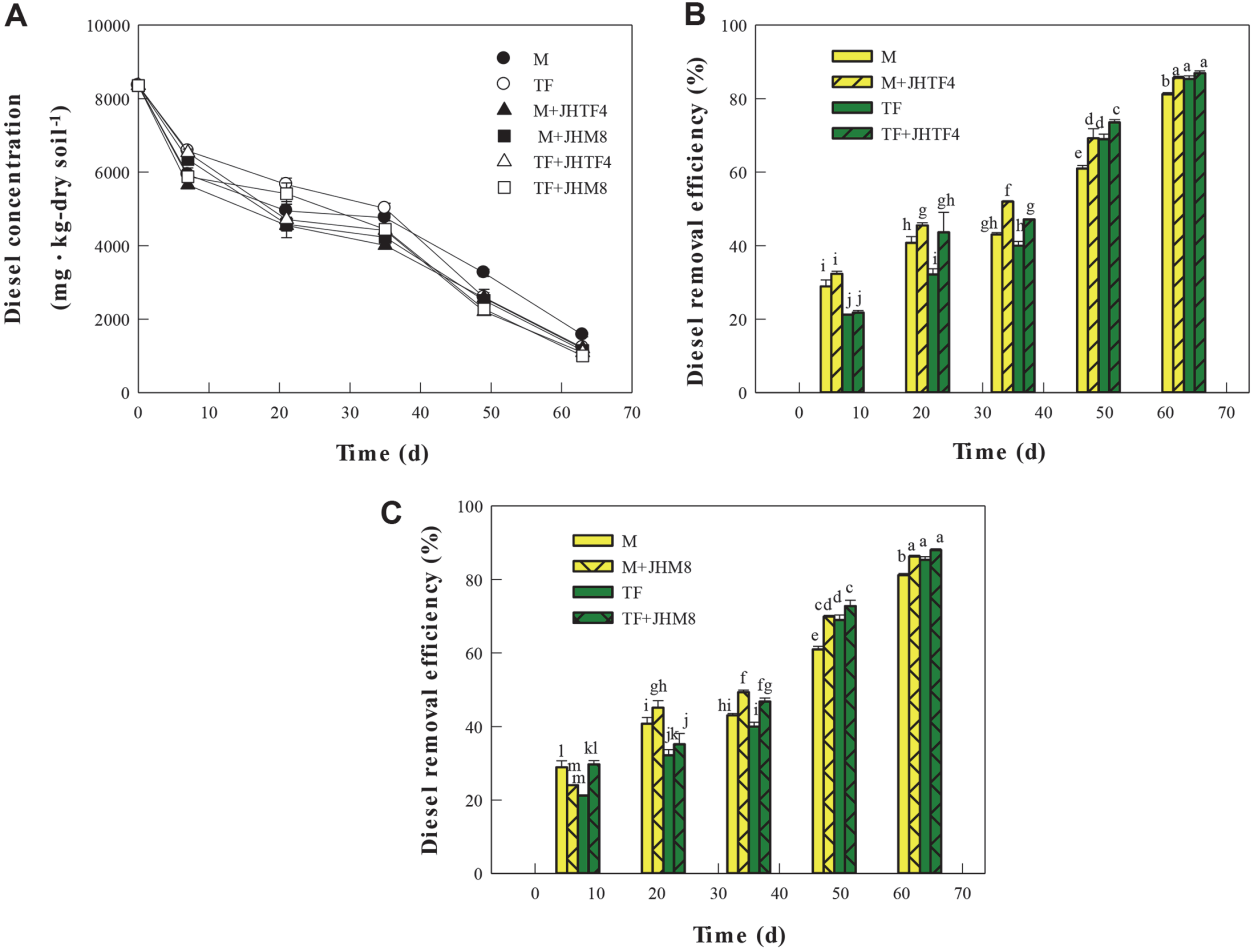
Effect of the inoculation of the isolates JHTF4 and JHM8 on diesel removal. (**A**) Residual diesel concentration in soil sample. (**B**) Diesel removal efficiency in the soil inoculated with JHTF4. (**C**) Diesel removal efficiency in the soil inoculated with JHM8. M, maize planting soil without inoculation; TF, tall fescue planting soil without inoculation; M+JHTF4, maize planting soil with JHTF4; M+JHM8, maize planting soil with JHM8; TF+JHTF4, tall fescue planting soil with JHTF4; TF+JHM8, tall fescue planting soil with JHM8. Different letters indicate significant differences between samples (*p* < 0.05).

To evaluate the inoculation effect of the strains JHTF4 and JHM8 on the diesel degradation, the residual TPH concentrations during rhizoremediation using maize and tall fescue were measured (Fig. 4A). Both strain JHTF4 and strain JHM8 couldn't degrade diesel (data not shown). In the soils planted with maize or tall fescue, TPH removal efficiencies with inoculating the strain JHTF4 were significantly higher than those without the inoculation until 50 days (Fig. 4B). At the 65th day, the TPH removal in the soil planted with maize and inoculated with strain JHTF4 was significantly higher than that without inoculation (Fig. 4B). However, in the soil planted with tall fescue, there was no significant difference in removal efficiency regardless of inoculation (Fig. 4B). The inoculation effect of strain JHM 8 on TPH removal showed a similar trend to that of strain JHTF4 (Fig. 4C). Until day 50, TPH removal efficiency was improved by strain inoculation in maize and tall fescue planting soil, but at day 63, TPH removal was improved by strain inoculation only in soil planted with maize.

Diesel, which contains hydrocarbons with approximately 12-20 carbon atoms, and diesel is known to be degraded by microorganisms with the *alkB* gene or with the CYP153 enzyme in aerobic environment [9, 42]. Some methylotrophs such as Methylophaga are known to have oil degrading ability [43], but the strains JHTF4 and JHM8 isolated in this study didn't have diesel-degrading ability. Therefore, the enhancement of diesel removal by inoculating the strains JHTF4 and JHM8 was not because they directly degraded diesel. Methanotrophs can utilize low-molecular hydrocarbon compounds and aromatic compounds as carbon sources [23, 44, 45]. Considering these capacity, the strains JHTF4 and JHM8 could improve diesel removal by playing a role in utilizing diesel-degrading intermediates that inhibit the activity of diesel-degrading bacteria during rhizoremediation process.

### Inoculation Effect of the Isolates JHTF4 and JHM8 on CH_4_ Oxidation Potential and *pmoA* Gene Dynamics in Soil

**Fig. 5:**
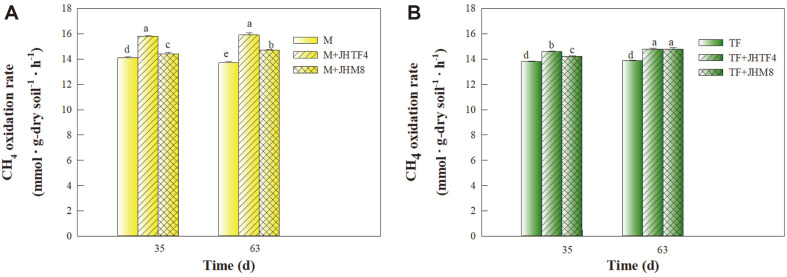
Comparison of soil CH_4_ oxidation rates in soil planted with maize (A) and tall fescue (B). M, maize planting soil without inoculation; M+JHTF4, maize planting soil with JHTF4; M+JHM8, maize planting soil with JHM8; TF, tall fescue planting soil without inoculation; TF+JHTF4, tall fescue planting soil with JHTF4; TF+JHM8, tall fescue planting soil with JHM8. Different letters indicate significant differences between samples (*p* < 0.05).

Fig. 5 shows the inoculation effect of the strains JHTF4 and JHM8 on the CH_4_ oxidation potential of rhizosphere soils planted with maize and tall fescue. In the soil planted with maize, the CH_4_ oxidation potentials of the inoculated soil with the strains JHTF4 and JHM8 were significantly higher than the potentials of the non-inoculated soil at 35th and 63th day (Fig. 5A). At 63th day, the CH_4_ oxidation potentials with the inoculation of the strains JHTF4 and JHM8 were 1.16 (15.9 ± 0.19 mmol CH_4_·g dry soil^−1^·h^−1^) and 1.07 (14.7 ± 0.07 mmol CH_4_·g dry soil^−1^·h^−1^) times of the potential of the non-inoculated soil (13.70 ± 0.1 mmol CH_4_·g dry soil^−1^·h^−1^), respectively. In the soil planted with tall fescue, the soil CH_4_ oxidation potentials were also significantly enhanced by inoculating with the strains JHTF4 and JHM8 (Fig. 5B). At 63th day, the CH_4_ oxidation potentials with the inoculation of the strains JHTF4 and JHM8 were 14.8 ± 0.07 and 14.8 ± 0.12 mmol CH_4_·g dry soil^−1^·h^−1^, while the CH_4_ oxidation potential of the non-inoculated soil was 13.90 ± 0.06 mmol CH_4_·g dry soil^−1^·h^−1^. These results suggest that the CH_4_-oxidizing activities of the strains JHTF4 and JHM8 displayed in the soil system, resulting in an improvement in soil CH_4_ oxidation potentials. CH_4_ is generally produced by methanogens in anaerobic zone of soil [46], but it is consumed by methanotrophs in aerobic zone of soil [47]. In addition, root-associated methanotrophs contribute in mitigating CH_4_ emission from the wetlands and soils [7, 20, 25, 47].

**Fig. 6:**
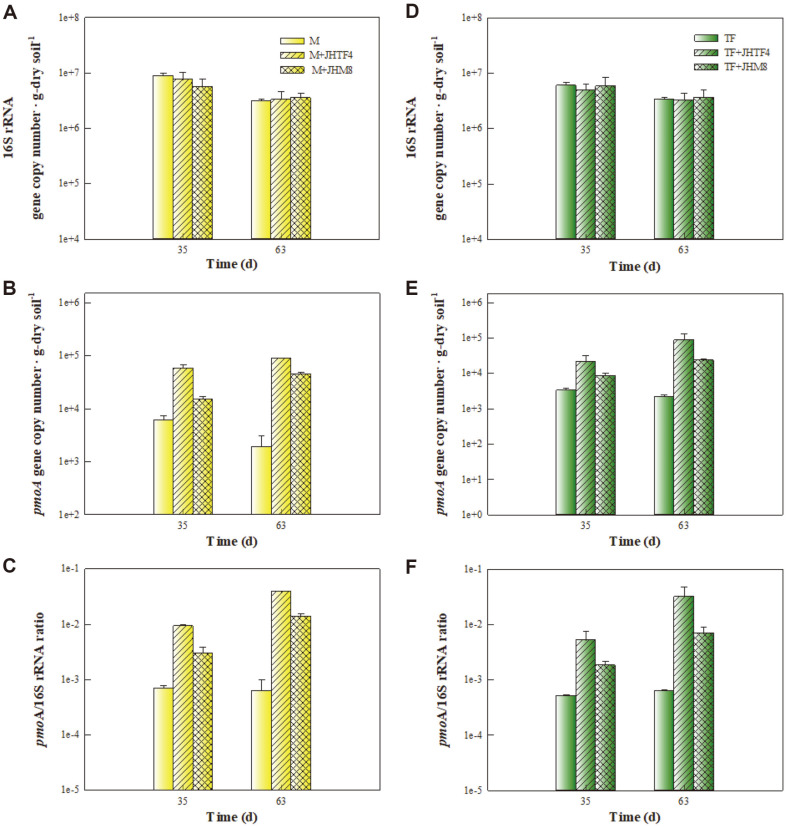
Comparison of gene copy numbers. (**A**) & (**D**) 16S rRNA gene copy number. (**B**) & (**E**) *pmoA* gene copy number. (**C**) & (**F**) *pmoA*/16S rRNA gene copy number ratio. M, maize planting soil without inoculation; M+JHTF4, maize planting soil with JHTF4; M+JHM8, maize planting soil with JHM8; TF, tall fescue planting soil without inoculation; TF+JHTF4, tall fescue planting soil with JHTF4; TF+JHM8, tall fescue planting soil with JHM8.

During rhizoremediation of diesel-contaminated soil, the inoculation effect of the strains JHTF4 and JHM8 on dynamics of the *pmoA* gene, which is responsible to CH_4_ oxidation [7, 20], is shown in Fig. 6. To compare total bacterial biomass in the soils without and with inoculation, 16S rRNA gene copy numbers were measured (Figs. 6A and 6D). 16S rRNA gene copy number weren't significantly different in all soils regardless of inoculation (3.1 × 10^6^ - 8.8 × 10^6^ copy number∙g-dry soil^-1^ in the maize planting soils and 3.3 × 10^6^ - 6.0 × 10^6^ copy number∙g-dry soil^-1^ in the tall fescue planting soils). However, in both the maize and tall fescue planting soils, the *pmoA* gene copy numbers in the inoculated soils were significantly higher than those in the non-inoculated soils (Figs. 6B and 6E). In the inoculated soils, the copy number ratio of *pmoA* gene to 16S rRNA gene (*pmoA*/16S rRNA ratio) tended to increase during rhizoremedition. In the maize planting soils with the strains JHTF4 and JHM8, the *pmoA*/16S rRNA ratios increased 4.2 and 4.7 times, respectively, from 35th day to 63th day. In addition, the *pmoA*/ 16S rRNA ratios also increased 6.3 and 3.8 times in the tall fescue planting soils with the strains JHTF4 and JHM8, respectively. CH_4_ oxidation rate increased with *pmoA* gene copy numbers in beech soil [48]. In addition, the *pmoA* gene copy number and CH_4_ oxidation activity tended to be proportional to each other in ammonium sulfate-added soil [49]. Soil CH_4_ oxidation potentials were closely associated with *pmoA* gene copy numbers during rhizoremediation of diesel-contaminated soil [7, 20]. Based on the results in Figs. 5 and 6, it can be confirmed that seen that *Methylocystis* sp. JHTF 4 and *Methyloversatilis* sp. JHM8, inoculated into the soils planted with maize or tall fescue, exhibits CH_4_ oxidation activity while surviving well in the soils.

## Conclusion

Considering global climate change by increasing greenhouse gas emissions, the innovation of rhizoremediation technology is required to mitigate CH_4_ emission during the biodegradation of petroleum hydrocarbons such as diesel and gasoline. In this study, two methanotrophs were isolated, and the inoculation effect of the isolates on diesel removal and soil CH_4_ oxidation potential was investigated during the rhizoremediation of diesel-contaminated soil planted with tall fescue (*F. arundinacea*) or maize (*Z. mays*). The isolate JHTF4 from the tall fescue rhizosphere and the isolate JHM8 from the maize rhizosphere were identified as *Methylocystis* sp. and *Methyloversatilis* sp., respectively. The maximum CH_4_ oxidation rates were 65.8 mmol·g-DCW^-1^·h^-1^ for the isolate JHTF4 and 33.8 mmol·g-DCW^-1^·h^-1^ for the isolate JHM8, which are superior to the rate for previously reported *Methylocystis* sp. When each isolate was added to diesel-contaminated soil planted with tall fescue or maize, the soil CH_4_ oxidation potentials as well as diesel removal efficiencies were significantly higher than those of the non-inoculated soils. Moreover, the *pmoA* gene copy numbers and the *pmoA*/16S rRNA ratios were also higher in the inoculated soils. Overall, these results suggest by inoculating *Methylocystis* sp. JHTF4 and *Methyloversatilis* sp. JHM8, not only the rhizoremediation performance can be improved, but also methane emissions can be reduced during the rhizoremediation of diesel-contaminated soil using tall fescue or maize. This study is the first report showing the possibility of reducing CH_4_ emissions and improving remediation efficiency by applying a pure methanotroph. Further studies are required to characterize the relationship among the inoculated methanotroph, indigenous soil bacteria and plant roots for the satisfactory remediation efficiency and CH_4_ mitigation during the rhizoremediation.

## References

[1] Saravanan A, Jeevanantham S, Narayanan VA, Kumar PS, Yaashikaa PR, Muthu CM (2020). Rhizoremediation - A promising tool for the removal of soil contaminants: a review. J. Environ. Chem. Eng..

[2] Syranidou E, Christofilopoulos S, Kalogerakis N (2017). *Juncus* spp.-The helophyte for all (phyto)remediation purposes?. N. Biotechnol..

[3] Gainer A, Bresee K, Hogan N, Siciliano SD (2019). Advancing soil ecological risk assessments for petroleum hydrocarbon contaminated soils in Canada: persistence, organic carbon normalization and relevance of species assemblages. Sci. Total Environ..

[4] Park IS, Park JW (2011). Determination of a risk management primer at petroleum-contaminated sites: developing new human health risk assessment strategy. J. Hazard. Mater..

[5] Baoune H, Aparicio JD, Acuña A, El Hadj-khelil AO, Sanchez L, Polti MA (2019). Effectiveness of the *Zea mays*-*Streptomyces* association for the phytoremediation of petroleum hydrocarbons impacted soils. Ecotoxicol. Environ. Saf..

[6] Lee YY, Lee SY, Lee SD, Cho KS (2022). Seasonal dynamics of bacterial community structure in diesel oil-contaminated soil cultivated with tall fescue (*Festuca arundinacea*). Int. J. Environ. Res. Public Health.

[7] Seo Y, Cho KS (2021). Effects of plant and soil amendment on remediation performance and methane mitigation in petroleumcontaminated soil. J. Microbiol. Biotechnol..

[8] Shahzad A, Siddiqui S, Bano A, Sattar S, Hashmi MZ, Qin M (2020). Hydrocarbon degradation in oily sludge by bacterial consortium assisted with alfalfa (*Medicago sativa* L.) and maize (*Zea mays* L.). Arab. J. Geosci..

[9] Correa-García S, Pande P, Séguin A, St-Arnaud M, Yergeau E (2018). Rhizoremediation of petroleum hydrocarbons: a model system for plant microbiome manipulation. Microb. Biotechnol..

[10] Jason-Ogugbue VT, Mmom PC, Etela I, Orluchukwu JA (2021). Uptake and bioaccumulation of diverse hydrocarbon compounds by selected food plants artificially exposed to bioremediated crude oil-contaminated soils. Acta Fytotechn Zootechn.

[11] Yang J, Li G, Qian Y, Zhang F (2018). Increased soil methane emissions and methanogenesis in oil contaminated areas. L. Degrad. Dev..

[12] IPCC (2014). Climate Change 2014: Synthesis Report.

[13] Ming A, Rowell I, Lewin S, Rouse R, Aubry T, Boland E (2021). Key messages from the IPCC AR6 climate science report. Cambridge Open Engage..

[14] Semple KT, Reid BJ, Fermor TR (2001). Impact of composting strategies on the treatment of soils contaminated with organic pollutants. Environ. Pollut..

[15] Davamani V, Parameswari E, Arulmani S (2020). Mitigation of methane gas emissions in flooded paddy soil through the utilization of methanotrophs. Sci. Total Environ..

[16] Lee YY, Jung H, Ryu HW, Oh KC, Jeon JM, Cho KS (2018). Seasonal characteristics of odor and methane mitigation and the bacterial community dynamics in an on-site biocover at a sanitary landfill. Waste Manag..

[17] Reis PCJ, Ruiz-González C, Crevecoeur S, Soued C, Prairie YT (2020). Rapid shifts in methanotrophic bacterial communities mitigate methane emissions from a tropical hydropower reservoir and its downstream river. Sci. Total Environ..

[18] Ruiz-Ruiz P, Gómez-Borraz TL, Revah S, Morales M (2020). Methanotroph-microalgae co-culture for greenhouse gas mitigation: effect of initial biomass ratio and methane concentration. Chemosphere.

[19] Yang H, Jung H, Oh K, Jeon JM, Cho KS (2021). Characterization of the bacterial community associated with methane and odor in a pilot-scale landfill biocover under moderately thermophilic conditions. J. Microbiol. Biotechnol..

[20] Lee YY, Seo Y, Ha M, Lee J, Yang H, Cho KS (2021). Evaluation of rhizoremediation and methane emission in diesel-contaminated soil cultivated with tall fescue (*Festuca arundinacea*). Environ. Res..

[21] Herlemann DP, Labrenz M, Jürgens K, Bertilsson S, Waniek JJ, Andersson AF (2011). Transitions in bacterial communities along the 2000 km salinity gradient of the Baltic Sea. ISME J..

[22] Kim TG, Lee EH, Cho KS (2011). Microbial community analysis of a methane-oxidizing biofilm using ribosomal tag pyrosequencing. J. Microbiol. Biotechnol..

[23] Lee EH, Yi TW, Moon KE, Park HJ, Ryu HW, Cho KS (2011). Characterization of methane oxidation by a methanotroph isolated from a landfill cover soil, South Korea. J. Microbiol. Biotechnol..

[24] Jäckel U, Schnell S, Conrad R (2004). Microbial ethylene production and inhibition of methanotrophic activity in a deciduous forest soil. Soil Biol. Biochem..

[25] Lee S, Kim S, Kim YJ, Lee Y, Cho KS (2021). Characterization of CH_4_-oxidizing and N_2_O-reducing bacterial consortia enriched from the rhizospheres of maize and tall fescue. Microbiol. Biotechnol. Lett..

[26] Kolb S, Knief C, Stubner S, Conrad R (2003). Quantitative detection of methanotrophs in soil by Novel. Society.

[27] Hanson RS, Hanson TE (1996). Methanotrophic bacteria. Microbiol. Rev..

[28] Abazari M, Owlia P, Zarrini G, Aghdasinia H (2021). Methane removal of isolated *Methylocystis* strains in the culture medium designed by evaluating strain capacity under adverse donditions. Biol. J. Microorg..

[29] Dunfield PF, Yimga MT, Dedysh SN, Berger U, Liesack W, Heyer J (2002). Isolation of a *Methylocystis* strain containing a novel *pmoA*like gene. FEMS Microbiol. Ecol..

[30] Jung GY, Rhee SK, Han YS, Kim SJ (2020). Genomic and physiological properties of a facultative methane-oxidizing bacterial strain of *Methylocystis* sp. from a wetland. Microorganisms.

[31] Rumah BL, Stead CE, Claxton Stevens BH, Minton NP, Grosse-Honebrink A, Zhang Y (2021). Isolation and characterisation of *Methylocystis* spp. for poly-3-hydroxybutyrate production using waste methane feedstocks. AMB Express.

[32] Pardi-Comensoli L, Tonolla M, Colpo A, Palczewska Z, Revikrishnan S, Heeb M (2022). Microbial depolymerization of epoxy resins: a novel approach to a complex challenge. Appl. Sci..

[33] Shrestha R, Černoušek T, Stoulil J, Kovářová H, Sihelská K, Špánek R (2021). Anaerobic microbial corrosion of carbon steel under conditions relevant for deep geological repository of nuclear waste. Sci. Total. Environ..

[34] Ossai IC, Ahmed A, Hassan A, Hamid F.S (2020). Remediation of soil and water contaminated with petroleum hydrocarbon: a review. Environ. Technol. Innov..

[35] Seo Y, Cho K (2020). Rhizoremdiation of petroleum hydrocarbon-contaminated soils and greenhouse gas emission characteristics: a Review. Microbiol. Biotechnol. Lett..

[36] de la Fuente Cantó C, Simonin M, King E, Moulin L, Bennett MJ, Castrillo G (2020). An extended root phenotype: the rhizosphere, its formation and impacts on plant fitness. Plant J..

[37] Koo SY, Cho KS (2006). Interaction between plants and rhizobacteria in phytoremediation of heavy metal-contaminated soil. Kor. J. Microbiol. Biotechnol..

[38] Vergani L, Mapelli F, Suman J, Cajthaml T, Uhlik O, Borin S (2019). Novel PCB-degrading *Rhodococcus* strains able to promote plant growth for assisted rhizoremediation of historically polluted soils. PLoS One.

[39] Iannucci A, Canfora L, Nigro F, De Vita P, Beleggia R (2021). Relationships between root morphology, root exudate compounds and rhizosphere microbial community in durum wheat. Appl. Soil Ecol..

[40] Hong SH, Ryu HW, Kim J, Cho KS (2011). Rhizoremediation of diesel-contaminated soil using the plant growth-promoting rhizobacterium *Gordonia* sp. S2RP-17. Biodegradation.

[41] Lee YY, Seo Y, Ha M, Lee J, Yang H, Cho KS (2021). Dynamics of bacterial functional genes and community structures during rhizoremediation of diesel-contaminated compost-amended soil. J. Environ. Sci. Heal. - Part A Toxic/Hazardous Subst. Environ. Eng..

[42] Nie Y, Chi CQ, Fang H, Liang JL, Lu SL, Lai GL (2014). Diverse alkane hydroxylase genes in microorganisms and environments. Sci. Rep..

[43] Gutierrez T, Aitken MD (2014). Role of methylotrophs in the degradation of hydrocarbons during the deepwater horizon oil spill. ISME J..

[44] Chen P, Liu H, Xing Z, Wang Y, Zhang X, Zhao T (2022). Cometabolic degradation mechanism and microbial network response of methanotrophic consortia to chlorinated hydrocarbon solvents. Ecotoxicol. Environ. Saf..

[45] Semrau JD (2011). Bioremediation via methanotrophy: overview of recent findings and suggestions for future research. Front. Microbiol..

[46] Serrano-Silva N, Sarria-Guzmán Y, Dendooven L, Luna-Guido M (2014). Methanogenesis and methanotrophy in soil: a review. Pedosphere.

[47] Cui J, Zhao J, Wang Z, Cao W, Zhang S, Liu J (2020). Diversity of active root-associated methanotrophs of three emergent plants in a eutrophic wetland in northern China. AMB Express.

[48] Degelmann DM, Borken W, Drake HL, Kolb S (2010). Different atmospheric methane-oxidizing communities in european beech and norway spruce soils. Appl. Environ. Microbiol..

[49] Alam MS, Jia Z (2012). Inhibition of methane oxidation by nitrogenous fertilizers in a paddy soil. Front. Microbiol..

